# Inseparable companions: Fungal viruses as regulators of fungal fitness and host adaptation

**DOI:** 10.3389/fcimb.2022.1020608

**Published:** 2022-10-13

**Authors:** Vanda Lerer, Neta Shlezinger

**Affiliations:** Koret School of Veterinary Medicine, The Robert H. Smith Faculty of Agriculture, Food and Environment, The Hebrew University of Jerusalem, Rehovot, Israel

**Keywords:** mycovirus, RCD, fungal virulence, host-pathogen interactions, symbiosis, multipartite interaction, host adaptation, fungal fitness

## Main

Viruses are unanimously omnipresent in the biosphere (Cobián Güemes et al., 2016). While fungi are a major cause of human, animal, and plant disease, they too can be infected by viruses. Mycoviruses are viruses that use the fungal molecular machinery for self-replication and sustainability (Son et al., 2015). Mycoviruses are widespread in the fungal kingdom, infecting over 20% of tested isolates. The vast majority of so-far identified mycoviruses have (+)ssRNA or dsRNA genome organization, with a smaller representation of (−)ssRNA and ssDNA viruses (reviewed in [Kondo et al., 2022)]. While some mycoviral infections are benign, there are examples of mycoviruses having both beneficial and detrimental effects on their fungal host, such as modified virulence (hypo or hyper virulence) (Melzer et al., 2002; Lau et al., 2018), mycotoxin production (Nerva et al., 2019), and adaptation to new environments (Nerva et al., 2017).

To date, there’s no evidence of a mycoviral lytic extracellular phase. While mycoviruses’ size ranges from 30-80 nm, the fungal cell wall pores only measure ~5.8 nm (De Nobel et al., 1990). Thus, the absence of an extracellular transmission route is attributable to the virtually impervious fungal cell wall (Kotta-Loizou and Coutts, 2017). For this reason, contact transmission is very rare, and mycoviruses transmission occurs chiefly vertically *via* sexual and asexual spore production and horizontally *via* cytoplasmic exchange following hyphal anastomosis thus, they generally rely on their fungal hosts for survival and proliferation (Kotta-Loizou and Coutts, 2017). One important caveat is that this transmission paradigm cannot explain the widespread prevalence and the frequent interspecies transmission between genetically incompatible groups (Melzer et al., 2002; Liu et al., 2003; Brasier et al., 2004; Ikeda et al., 2005; Charlton et al., 2008). Perhaps, mycoviruses can be spread by contact between wounded fungal cells, or by insect vectors that can breach the fungal cell wall, similar to plant viruses. As a precedent for this proposition, *Sclerotinia sclerotiorum hypovirulence-associated DNA virus 1 (SsHADV-1)* uses the mycophagous insect *Lycoriella ingenua* as a transmission vector (Liu et al., 2016).

This key feature of mycoviruses, namely the lack of extracellular phase, resembles the lifestyle of lysogenic or latent infections, which transmit vertically from generation to generation (Feiner et al., 2015). This phenomenon generates predation pressure since mycoviral reproductive success is intimately linked to that of their fungal hosts. Fungal acquisition of an anti-mycoviral defense mechanism may demolish the mycovirus, and conversely, an increased mycoviral virulence risks the extinction of the fungal host. With this in view, it is to be expected that mutualistic interactions that support both mycoviral and fungal reproduction have evolved. Pathogenic fungi need to survive extremely inhospitable environments such as oxidative burst (plants and phagocytes), and elevated body temperatures that are known to induce genetically regulated cell death (RCD) and stress pathways in fungal cells (Shlezinger et al., 2011a; Shlezinger et al., 2017). In this context, we predict that symbiotic mycoviruses that aim to keep their hosts alive will strive to prevent the induction of fungal cell death by targeting fungal stress and survival pathways, thereby governing fungal virulence and adaptation to new environments and new hosts.

In this opinion article, we focus on favorable mycovirus-fungus interactions and propose that mycoviruses hijack fungal survival pathways and thereby modify fungal virulence and host adaptation. These mycovirus-derived traits that enhance fungal fitness in specialized niches underlie fungal virulence and adaptation and may account for host-jumps and crossover events.

## Microbial hacking: Stress and cell death pathways in virus-host interactions

Control of cell death pathways plays a central role in immunity to infection in animals and plants, through the elimination of infected host cells, or extension of host cell survival, depending upon the pathogens’ infection strategy (Coll et al., 2011; Jorgensen et al., 2017). In turn, numerous microbes have evolved evasion strategies to modulate host cell death and survival pathways, to generate replicative niches, or to facilitate tissue invasion (Navarre and Zychlinsky, 2000).

In the broadest sense, viruses can be categorized according to their pathogenic lifestyle into lytic and latent viruses. During the lytic phase, viruses enter a productive cycle leading to virion release by cell lysis, while latent viruses persist within the host cell. Viruses have evolved to manipulate an array of metabolic and survival mechanisms to their advantage by inducing or preventing cell death, depending on their infection strategy (Benedict et al., 2002). Accordingly, latent viruses often prevent the death of the host cell to establish a suitable environment for long-term persistence (Connolly and Fearnhead, 2017). Viruses employ diverse strategies including viral mimicry and epigenetic reprogramming, to rewire host cellular networks [reviewed in (Davey et al., 2011)]. A well-studied example is the *Epstein–Barr virus (EBV)* latent membrane protein 1 (LMP1) which is a constrictively-expressed mimetic of host receptor CD40 that mediates the activation of nuclear factor-kappa B (NF-κB) signaling, resulting in RCD blockage and cell proliferation (Thorley-Lawson, 2001).

**Figure 1:**
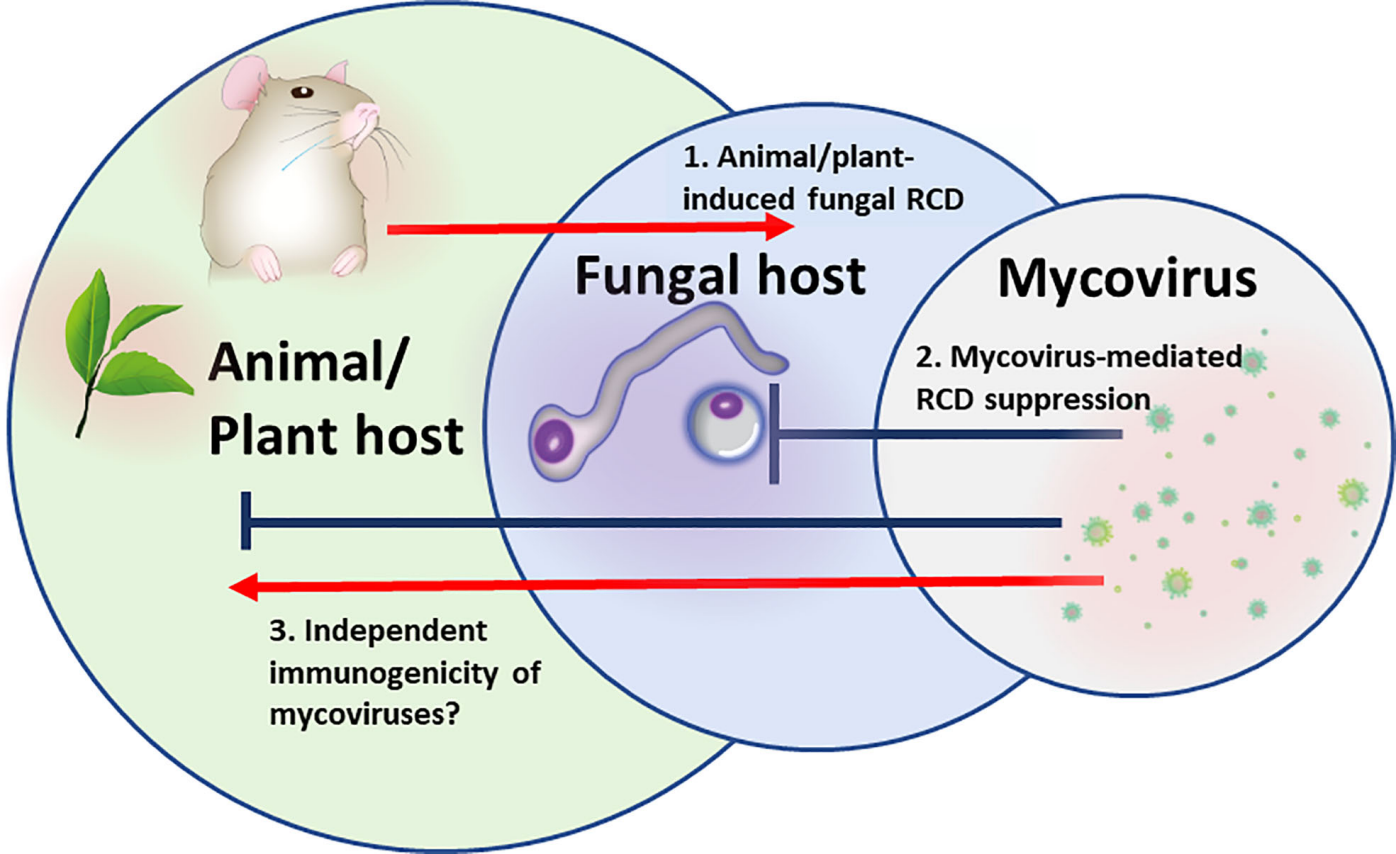
The “Russian doll” model of multipartite RCD-driven pathosystem: Mycovirus, fungal pathogen, animal/plant host. 1. Animal/plant host induction of regulated cell death (RCD) pathways in the fungus to eliminate fungal spread. 2. Mycoviral-mediated inhibition of RCD or activation of survival mechanisms in fungal cells to prevent fungal host cells from dying. 3.Mycoviral immunomodulation. Mycoviral proteins may serve as PAMPs sensed by animal/plant cell PRRs and directly stimulate or block multiple inflammatory and cell death signaling pathways.

In multipartite “Russian doll” pathosystems in which a virus is infecting a pathogen, infecting an animal or a plant (virus- microbial host-multicellular host), the fungal pathogen has to survive within the hostile host environment (**Figure 1**). Both, plant and animal defenses generate a robust oxidative burst and antimicrobial peptides that have the potential to induce microbial cell death, and particularly RCD (Madeo et al., 1999; Andres et al., 2008; Shlezinger et al., 2011b). Under these circumstances, the fungal pathogen will benefit from the presence of latent/chronic viruses that provide competitiveness by fortifying stress resistance.

Accordingly, the selective pressure imposed by the animal/plant host serves as a vital factor for maintaining viral latency. Indeed, while very prevalent in pathogenic interactions, biofilms, and harsh environments, lysogeny-derived traits of phages are dispensable under standard laboratory conditions (Wang et al., 2010). Cryptic lysogens bestow a wealth of beneficial traits to their bacterial hosts such as enhanced antibiotic resistance, oxidative stress, and osmotic resistance (to name a few) (Wang et al., 2010). The bacterial host reprogramming is mediated, at least in part, by the inhibition of bacterial pro-death modules (Engelberg–Kulka et al., 1998; Engelberg-Kulka and Kumar, 2015). 

## Mycoviral infection and its numerous fitness benefits

Similar to lysogenic or chronic phages, mutually beneficial interactions that support mycoviral propagation and fungal fitness have evolved. The most well-studied favorable interaction is the killer phenotype conferred by mycoviral-encoded secreted toxins, similar to phage-derived bacterial toxins such as diphtheria, botulinum, and cholera (Freeman, 1951; Barksdale and Arden, 1974; Waldor and Mekalanos, 1996). This mutualistic interaction leads to the production of a toxin that kills susceptible competitors (Allen et al., 2013; McBride et al., 2013).

Recent studies suggest that similar to mammalian viruses and bacteriophages, mycoviruses may control the survival and fate of their fungal host by modulating stress and RCD pathways to suit their needs and complete their replication cycle. For instance, MrV40, a dsRNA mycovirus infecting *Malassezia* species, was shown to upregulate host genes that are involved in RCD (Park et al., 2020). This includes *HOG1* and *ATG101* (osmotic stress and autophagy-related proteins). Moreover, Clancey et al., 2020 reported enhanced colonization by MsMV1-containing *Malassezia* isolates in an epicutaneous murine model. Similarly, *Talaromyces marneffeipartitivirus-1* (*TmPV1*) is highly abundant in clinical *T. marneffei* isolates and the mycoviral infection exacerbates disease in a murine systemic talaromycosis model. While the underlying mechanism is yet to be clarified, *TmPV1* is associated with upregulation of potential virulence factors (GABA transaminase, nitrate transporter, nitrite reductase) and downregulation of RNAi-related genes (Lau et al., 2018). White-nose syndrome (WNS) is a devastating fungal disease that is killing off the bat population of North America (Blehert et al., 2009). The microbial culprit is the mycovirus-infected fungus *Pseudogymnoascus destructans*, harboring the *Pseudogymnoascus destructans partitivirus* (*PdPV*). *PdPV* is associated with *P. destructans* strains accountable for WNS, and was shown to confer *P. destructans* a growth advantage on a porcine ear model (Thapa et al., 2016; Thapa et al., 2022). Conversely, *Sclerotinia sclerotiorum hypovirulence-associated DNA virus 1* (*SsHADV-1*) can modulate the virulence of its phytopathogenic fungal host *S. sclerotiorum* converting its pathogenic lifestyle from necrotrophic to endophytic. The observed endophytic conversion is associated with downregulation of plant cell wall-degrading enzymes (Zhang et al., 2020). Some mycoviruses confer enhanced tolerance to abiotic stresses that are known to induce fungal RCD. The dsRNA virus *Curvularia thermal tolerance virus* (*CThTV*) bestows its fungal host *Curvularia protuberate* and its host plant enhanced thermal protection (Márquez et al., 2007). Furthermore, infection of the phytopathogenic fungus *Cryphonectria parasitica* with *Penicillium aurantiogriseum partiti-like virus 1* (*PaPLV1*) results in improved osmotic stress tolerance (Huh et al., 2002; Nerva et al., 2017). These studies support the notion that mycoviral infections account for strain-specific virulence and host adaptation.

Similar to animal, plant, and bacterial innate immune systems, fungi have evolved a non-self surveillance system termed heterokaryon incompatibility (HI) to eliminate cytoplasmic mixing and transmission of mycoviruses and other deleterious cellular components upon hyphal anastomosis (Saupe, 2000). Allorecognition in filamentous fungi is genetically controlled and relies on the induction of spatiotemporally localized RCD upon fusion events between genetically incompatible strains determined by the *het* loci. The *het* genes are highly polymorphic belonging to the NOD-like receptors (NLRs) family. Animal and plant NLRs are intracellular nucleotide-binding site and leucine-rich repeats containing proteins controlling cell survival in response to immunogenic cues. Thus, HI may represent an ancestral form of allorecognition and antimicrobial innate immunity. The ultimate goal of a dweller mycovirus is the subversion of HI to prevent the host cell from dying and to facilitate horizontal transmission by hyphal fusion. Indeed *Sclerotinia sclerotiorum mycoreovirus 4* (*SsMYRV4*) suppresses HI-induced RCD in vegetatively incompatible S. *sclerotiorum* enabling heterologous mycoviral transmission by downregulating G proteins het-domain related genes (Wu et al., 2017).

## Mycoviruses: Backseat drivers of fungal disease 

To add another layer of complexity, microbial viruses of bacteria, protozoa, and fungi, have the potential to independently be immunogenic and stimulate multiple inflammatory, cell death, and survival signaling pathways in the animal or plant host. The immune system can be exposed to viral components upon intracellular killing and degradation of the carrier pathogen followed by recognition by pathogen recognition receptors (PPRs). Endosomal TLRs recognize nucleic acid motifs, with TLR9 recognizing DNA, TLR7 ssRNA, and TLR3 dsRNA (Doyle and O’Neill, 2006). Intracellular RNA sensing is mediated by cytosolic RIG-I–like receptors (RLR), composed of RIG-I, NLRs, and MDA5, that work through the MAVS adaptor to activate IFN antiviral responses (Rehwinkel and Gack, 2020). These pro-inflammatory pathways can often unleash cell death machinery. For instance, virus-stimulated TLR3 and TLR7 can trigger autophagy (a form of RCD) by shifting the balance of MyD88 and/or TRIF adaptor proteins interaction toward Beclin-1 and reducing the binding of Beclin-1 to the anti-apoptotic protein Bcl-2 (Delgado et al., 2008; Shi and Kehrl, 2008). TLR3/4 recruitment of TIR domain-containing adapter inducing IFN-β (TRIF) results in caspase 8-mediated apoptotic cell death (Ruckdeschel et al., 2004). Moreover, cytoplasmic recognition of viral RNA may trigger the formation of NLRP3 inflammasomes leading to host cell death by pyroptosis (Kanneganti et al., 2006). While such interactions are established for phages (Pajtasz-Piasecka et al., 2008; Kurzępa et al., 2009; Huh et al., 2019), information on non-bacterial microbial viruses is sparse. The dsRNA virus Leishmaniavirus (LRV), a parasite of *Leishmania* spp., is among the few known cases of non-bacterial microbial-virus that drives human disease. LRV is a potent innate immunogen, stimulating a detrimental TLR3-mediated hyperinflammatory response, and exacerbating disease (Ives et al., 2011; de Carvalho et al., 2019). Precedence for mycoviral immunogenicity was demonstrated for two mycoviruses of *Malassezia* spp. Showing TLR3- dependent (Park et al., 2020) and independent (Clancey et al., 2020) elevated type-I and II interferon expression.

Given the commonalities between known oncogenes and viral-targeted cellular pathways, it is not surprising that some mammalian viruses can cause cancer by circumventing or hijacking cell death modalities. Interestingly, a recent study discovered that plasma from lymphoblastic leukemia (ALL) patients, was reactive with supernatants from a mycovirus-containing *Aspergillus flavus* isolate, while sera from healthy controls were non-reactive, suggesting mycovirus-mediated leukemogenesis in this group of patients (Tebbi et al., 2021). The question arises, whether mycoviruses may impact human or animal health directly or once internalized using the fungal cell as a carrier.

## Concluding remarks

So far, the study of mycoviruses has been dominated by the search for pathogenic viruses, but this will need to change if we are to appreciate the diverse ways that viruses affect life on earth. It is becoming apparent that mycoviruses play a prominent role in various facets related to fungal virulence. This notion extends beyond the outlined examples and may represent an Achilles’ heel for many pathogens. Accordingly, the fungal and mammalian hosts’ anti-viral pathways may be instrumental in regulating fungal virulence and pathogenesis. Moreover, because stress and RCD pathways are likely to play a central role in all types of fungus–mycovirus interactions, key players identified in these pathways bear a substantial therapeutic potential, providing new targets that operate in an entirely unexploited target space. As fungal genomes are treasure troves of untapped compounds, further exploration of the molecular mechanisms governing mycoviral-mediated fungal fitness and virulence may lead to the development of novel viral-selective, pan-antiviral drugs.

## Author contributions

NS and VL designed and wrote this opinion. All authors contributed to the article and approved the submitted version.

## Funding

NS is generously supported by the Israeli Science Foundation (Grant # 1760/20), the Zuckerman STEM Leadership Program, the Ministry of Science and Technology of Israel (Grant #0001998), and BARD US-Israel Agricultural Research and Development Fund (Grant #IS-5492-22 R).

## Conflict of interest

The authors declare that the research was conducted in the absence of any commercial or financial relationships that could be construed as a potential conflict of interest.

## Publisher’s note

All claims expressed in this article are solely those of the authors and do not necessarily represent those of their affiliated organizations, or those of the publisher, the editors and the reviewers. Any product that may be evaluated in this article, or claim that may be made by its manufacturer, is not guaranteed or endorsed by the publisher.
